# The Microaerophilic Microbiota of *De-Novo* Paediatric Inflammatory Bowel Disease: The BISCUIT Study

**DOI:** 10.1371/journal.pone.0058825

**Published:** 2013-03-12

**Authors:** Richard Hansen, Susan H. Berry, Indrani Mukhopadhya, John M. Thomson, Karin A. Saunders, Charlotte E. Nicholl, W. Michael Bisset, Sabarinathan Loganathan, Gamal Mahdi, Dagmar Kastner-Cole, Andy R. Barclay, Jon Bishop, Diana M. Flynn, Paraic McGrogan, Richard K. Russell, Emad M. El-Omar, Georgina L. Hold

**Affiliations:** 1 Gastrointestinal Research Group, Division of Applied Medicine, University of Aberdeen, Foresterhill, Aberdeen, United Kingdom; 2 Child Health, University of Aberdeen, Royal Aberdeen Children’s Hospital, Foresterhill, Aberdeen, United Kingdom; 3 Department of Paediatric Gastroenterology, Royal Aberdeen Children’s Hospital, Foresterhill, Aberdeen, United Kingdom; 4 Department of Paediatrics, Ninewells Hospital and Medical School, Dundee, United Kingdom; 5 Department of Paediatric Gastroenterology, Royal Hospital for Sick Children, Glasgow, United Kingdom; Charité-University Medicine Berlin, Germany

## Abstract

**Introduction:**

Children presenting for the first time with inflammatory bowel disease (IBD) offer a unique opportunity to study aetiological agents before the confounders of treatment. Microaerophilic bacteria can exploit the ecological niche of the intestinal epithelium; *Helicobacter* and *Campylobacter* are previously implicated in IBD pathogenesis. We set out to study these and other microaerophilic bacteria in *de-novo* paediatric IBD.

**Patients and Methods:**

100 children undergoing colonoscopy were recruited including 44 treatment naïve *de-novo* IBD patients and 42 with normal colons. Colonic biopsies were subjected to microaerophilic culture with Gram-negative isolates then identified by sequencing. Biopsies were also PCR screened for the specific microaerophilic bacterial groups: Helicobacteraceae, Campylobacteraceae and *Sutterella wadsworthensis.*

**Results:**

129 Gram-negative microaerophilic bacterial isolates were identified from 10 genera. The most frequently cultured was *S. wadsworthensis* (32 distinct isolates). Unusual *Campylobacter* were isolated from 8 subjects (including 3 *C. concisus*, 1 *C. curvus*, 1 *C. lari*, 1 *C. rectus*, 3 *C. showae*). No *Helicobacter* were cultured. When comparing IBD vs. normal colon control by PCR the prevalence figures were not significantly different (*Helicobacter* 11% vs. 12%, p = 1.00; *Campylobacter* 75% vs. 76%, p = 1.00; *S. wadsworthensis* 82% vs. 71%, p = 0.312).

**Conclusions:**

This study offers a comprehensive overview of the microaerophilic microbiota of the paediatric colon including at IBD onset. *Campylobacter* appear to be surprisingly common, are not more strongly associated with IBD and can be isolated from around 8% of paediatric colonic biopsies. *S. wadsworthensis* appears to be a common commensal. *Helicobacter* species are relatively rare in the paediatric colon.

**Trial Registration:**

This study is publically registered on the United Kingdom Clinical Research Network Portfolio (9633).

## Introduction

Paediatric inflammatory bowel disease (IBD) represents a variant phenotype characterised by more extensive disease activity at onset and a progressive course [1]. Immunological differences can be identified between paediatric and adult Crohn’s disease (CD) [2]. While paediatric disease represents a distinct phenotype of IBD, it is surprisingly not explained by a significantly different genotype [3]. One implication might be that the paediatric phenotype is an expression of different environmental triggers rather than inherited factors. Recent studies showing a rise in the incidence of IBD in childhood and, perhaps more worryingly, a younger age at onset in those affected support an urgent need for aetiological studies to explain these trends [4]–[7]. The discovery that the use of antibiotics early in life and in multiple courses confers an increased risk of subsequent IBD development demonstrates the importance of microbial perturbation in disease development [8], [9]. Recent genetic discoveries reinforce the essential role for host defence against infection in IBD pathogenesis [10].

The biological importance of the gastrointestinal microbiota and its symbiotic relationship with the human host is now firmly established [11], [12]. It is increasingly clear that disturbance of the resident microbiota can induce human disease, with the most studied example being the “dysbiosis” of IBD and its resultant inflammation [13], [14]. The route from health to IBD through dysbiosis is unclear but may involve a trigger event such as bacterial infection [15], [16]. We recently postulated that Proteobacteria with adherent and invasive properties may exploit weaknesses in host defences to drive this dysbiotic change [16]. *Helicobacter* species (microaerophilic members of the Epsilonproteobacteria; “microaerophilic” describing bacteria that thrive in low oxygen concentrations) have been shown to initiate IBD in both rodent and primate models and may also be implicated in infectious proctitis in humans [17]. Conflicting evidence exists from human studies to support *Helicobacter* as agents in human IBD [18]–[26]; nevertheless the compelling animal data has made the genus worthy of consideration as a potential pathogen in IBD. *Campylobacter concisus* (another microaerophilic Epsilonproteobacterium) was cultured from mucosal biopsies from a paediatric CD patient by Zhang *et al* [27]. This organism has since been shown to be more prevalent in IBD and to be capable of adhering to and invading epithelial cells and driving a pro-inflammatory change [28]–[31].

Much of the current literature on IBD microbiology utilises convenient cohorts of patients with established disease, potentially introducing major confounders when interpreting results [32]. Paediatric IBD offers an opportunity to explore these problems, since children are relatively free of additional significant co-morbidities and are generally treatment naïve at IBD diagnosis. For these reasons we set up the “Bacteria in Inflammatory bowel disease in Scottish Children Undergoing Investigation before Treatment” (BISCUIT) study, with the specific aims of:

1. Recruiting a robustly described, prospective clinical cohort of newly presenting children with untreated IBD alongside children with normal colons as controls
2. Isolating and identifying microaerophilic bacteria (particularly *Helicobacter* and *Campylobacter*) that may be of clinical relevance at the onset of IBD
3. Confirming the true prevalence of specific microaerophilic organisms within the colonic mucosa by molecular methods.

The BISCUIT study recruited 100 Scottish children over a 30 month period. The data contained within this paper documents the isolation and identification of microaerophilic bacteria alongside the molecular (true) prevalence of *Helicobacter and Campylobacter* species and *Sutterella wadsworthensis* within colonic biopsies from the entire BISCUIT cohort. In a complementary but distinct analysis we previously published a full hypothesis-free bacterial diversity assessment using pyrosequencing on a subset of the cohort (37 BISCUIT subjects in total) [32].

## Methods

Patients were recruited to the BISCUIT study from elective colonoscopy lists in three paediatric centres (Royal Aberdeen Children’s Hospital, Aberdeen; Royal Hospital for Sick Children, Glasgow and Ninewells Hospital, Dundee). An approach with study information was made either on the day of admission (the day before endoscopy) or by post in advance of admission. Patients were excluded if they received systemic antibiotics or steroids 3 months prior to their colonoscopy, immunosuppression at any time, or if they had a previous IBD diagnosis. IBD investigations were as per the Porto criteria. IBD diagnosis and phenotype were assigned with reference to the Lennard-Jones, Montreal and Paris criteria (Table S2) [33]–[36]. Comprehensive clinical data were also collected at recruitment by a single investigator through use of a standardised verbal questionnaire.

Initial recruitment was into two macroscopically-defined categories, those with likely IBD, at first presentation, with macroscopic colonic inflammation and those undergoing colonoscopy who subsequently had a normal colon macroscopically. Final diagnosis and disease categorisation was assigned once endoscopic, histological and radiological investigations were complete after a minimum of six months follow-up.

### Ethics Statement

Ethical approval was granted by North of Scotland Research Ethics Service (09/S0802/24) on behalf of all participating centres and written informed consent was obtained from the parents of all subjects. Informed assent was also obtained from older children who were deemed capable of understanding the nature of the study.

This study is publically registered on the United Kingdom Clinical Research Network Portfolio (9633).

Biopsies were taken from a single site, from the distal colon in controls (rectum/sigmoid) or from the most distal inflamed site in those with colonic inflammation. 5–6 biopsies were collected using standard endoscopic forceps from all recruits. 1–2 biopsies were used for microaerophilic culture work by transferring these biopsies immediately into individual 2 ml screw-top containers with ∼700 µl Brucella broth which were incubated at room temperature until plated. 2–3 biopsies were collected for DNA analysis into a sterile 1.5 ml Eppendorf container and placed immediately onto ice before transfer to −80°C storage. The remaining biopsy was collected in paraformaldehyde for future fluorescent *in-situ* hybridisation studies.

Culture work was performed as described in Mukhopadhya *et al* [37] utilising five selective plates and one plain blood agar plate, each incubated in microaerophilic gas conditions generated by Anoxomat® (Mart® Microbiology, Drachten, Netherlands) and reviewed twice weekly for up to one month. Gram-negative and oxygen sensitive (by virtue of failed subculture in room air) bacterial isolates were identified by sequencing of the 16S rRNA gene. A minimum read length of 400 bp was obtained for attributing bacterial identities, the result of which was searched against the NCBI BLAST database (http://blast.ncbi.nlm.nih.gov/Blast.cgi).

DNA extraction of mucosal biopsies was performed using the commercially available Qiagen QIAamp Mini kit (Qiagen, Crawley, UK) with minor modifications as described previously [25]. A test polymerase chain reaction (PCR) with biopsy DNA was performed utilising universal bacterial primers to confirm the suitability of the DNA for further analysis (**Table S1**) [38]. Conventional PCR was undertaken to determine the prevalence of *Helicobacter* genus, *Campylobacter* genus and *Sutterella wadsworthensis* using primers and conditions described previously (**Table S1**) [25], [30], [37]. PCR products from *Helicobacter* and *Campylobacter* genus reactions were either directly sequenced on an Applied Biosystems model 3730 automated capillary DNA sequencer or cloned first into JM109 competent cells with pGEM-T-easy vector if sequence analyses indicated a mixed sequencing profile [30].

*Helicobacter pylori* serology was performed using the Premier *H. pylori* enzyme immunoassay which detects IgG antibody (Meridian Bioscience).

All statistical comparisons were undertaken using SPSS Statistics version 20 (IBM Software 2010).

## Results

**Figure 1:**
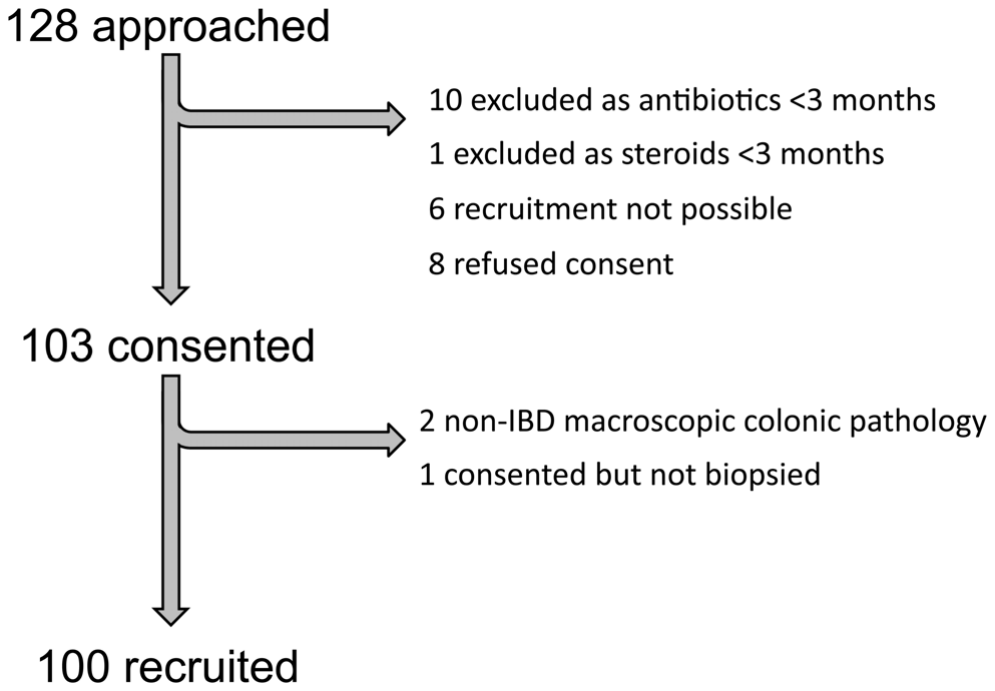
Recruitment flowchart of recruits to BISCUIT study. Those where recruitment was not possible were approached by post but could not then be recruited on their day of colonoscopy. The one child consented but not biopsied was due to unavailability of the investigator on the day in question.

128 Scottish children were approached for the study with 100 being recruited (**Figure 1**). Final categorisation was based on a thorough review of macroscopic, microscopic and available radiological data and is presented in **Table 1** and **Table 2** alongside categorical, demographic and numerical clinical data respectively. 44 IBD subjects were diagnosed as CD (29), ulcerative colitis (UC) (13) and IBD-type unclassified (IBD-U) (2). Individual IBD phenotypes are shown in **Table S2**. Granulomata were identified in at least one biopsy site in 21 of 29 (72.4%) CD recruits. A priori, the intention was to compare IBD recruits against those with a normal colon, however in order to achieve this, those with microscopic pathology were further sub-categorised. “Normal colon control” subjects had both a macroscopically and microscopically normal colon. “Eosinophilic controls” had histologically significant eosinophilic infiltration of their colonic mucosa in at least one site. “Non-specific inflamed, non-IBD” subjects had microscopic evidence of inflammation but insufficient grounds for an IBD diagnosis. The single “proto-IBD” recruit would have been classified within the “eosinophilic control” category but has since been re-evaluated and has subsequently developed CD. Data from each of these latter three phenotypic groups are presented in full; however statistical analyses compare the IBD and normal colon control groupings only.

**Table 1:** BISUIT Study Categorical Clinical Data.

	Eosinophilic control	IBD	*Crohn’s disease*	*IBD-type unspecified*	*Ulcerative colitis*	Non-specific inflamed non-IBD	NormalColoncontrol	Proto-IBD	AllRecruits	IBD vs. Normal Colon ControlFisher’s Exact Test (2-sided)
Total number	7	44	*29*	*2*	*13*	6	42	1	100	
Male (%)	3 (42.9)	30 (68.2)	*20 (69.0)*	*1 (50)*	*9 (69.2)*	2 (33.3)	33 (78.6)	1 (100)	69 (69.0)	0.334
Concurrent upper endoscopy (%)	6 (85.7)	**44 (100)**	*29 (100)*	*2 (100)*	*13 (100)*	2 (33.3)	**35 (83.3)**	1 (100)	88 (88.0)	**0.005**
Evidence of histological gastritis(% of upper endoscopies)	4 (66.7)	**38 (86.4)**	*27 (93.1)*	*1 (50.0)*	*10 (76.9)*	0 (0)	**12 (34.3)**	1 (100)	55 (62.5)	**<0.001**
*H. pylori* on histology(% of upper endoscopies)	1 (16.7)	**0 (0)**	*0 (0)*	*0 (0)*	*0 (0)*	0 (0)	**4 (11.4)**	0 (0)	5 (5.7)	**0.035**
**Symptoms over duration of illness (yes/no)**										
Abdominal Pain (%)	7 (100)	35 (79.5)	*23 (79.3)*	*2 (100)*	*10 (76.9)*	4 (66.7)	36 (85.7)	0 (0)	82 (82.0)	0.573
Diarrhoea (%)	5 (71.4)	**37 (84.1)**	*23 (79.3)*	*2 (100)*	*12 (92.3)*	3 (50.0)	**25 (59.5)**	1 (100)	71 (71.0)	**0.016**
Tenesmus (%)	4 (57.1)	30 (68.2)	*18 (62.1)*	*1 (50.0)*	*11 (84.6)*	2 (33.3)	24 (57.1)	1 (100)	61 (61.0)	0.373
Blood in stool (%)	5 (71.4)	31 (70.5)	*16 (55.2)*	*2 (100)*	*13 (100)*	6 (100)	24 (57.1)	1 (100)	67 (67.0)	0.262
Constipation (%)	2 (28.6)	16 (36.4)	*12 (41.4)*	*0 (0)*	*4 (30.8)*	3 (50.0)	11 (26.2)	0 (0)	32 (32.0)	0.358
Blood on wiping bottom (%)	6 (85.7)	**29 (65.9)**	*17 (58.6)*	*1 (50.0)*	*11 (84.6)*	5 (83.3)	**17 (40.5)**	1 (100)	58 (58.0)	**0.03**
Anorexia (%)	3 (42.9)	**31 (70.5)**	*22 (75.9)*	*1 (50.0)*	*8 (61.5)*	1 (16.7)	**14 (33.3)**	0 (0)	49 (49.0)	**0.001**
Nausea (%)	3 (42.9)	17 (38.6)	*14 (48.3)*	*0 (0)*	*3 (23.1)*	2 (33.3)	14 (33.3)	1 (100)	37 (37.0)	0.658
Vomiting (%)	3 (42.9)	10 (22.7)	*8 (27.6)*	*0 (0)*	*2 (15.4)*	1 (16.7)	5 (11.9)	1 (100)	20 (20.0)	0.258
Heartburn (%)	0 (0)	7 (15.9)	*5 (17.2)*	*0 (0)*	*2 (15.4)*	0 (0)	10 (23.8)	0 (0)	17 (17.0)	0.423
Parentally reported weight loss (%)	1 (14.3)	25 (56.8)	*18 (62.1)*	*0 (0)*	*7 (53.8)*	0 (0)	19 (45.2)	0 (0)	45 (45.0)	0.388
Parentally reported poor growth (%)	2 (28.6)	7 (15.9)	*5 (17.2)*	*1 (50.0)*	*1 (7.7)*	0 (0)	4 (9.5)	0 (0)	13 (13.0)	0.522
**Comorbidities**										
Asthma (%)	3 (42.9)	10 (22.7)	*7 (24.1)*	*1 (50.0)*	*2 (15.4)*	1 (16.7)	6 (14.3)	1 (100)	21 (21.0)	0.409
Eczema (%)	1 (14.3)	10 (22.7)	*9 (31.0)*	*1 (50.0)*	*0 (0)*	1 (16.7)	6 (14.3)	1 (100)	19 (19.0)	0.409
Hayfever (%)	3 (42.9)	13 (29.5)	*8 (27.6)*	*2 (100)*	*3 (23.1)*	1 (16.7)	5 (11.9)	0 (0)	22 (22.0)	0.063
Allergies (%)	3 (42.9)	8 (18.2)	*6 (20.7)*	*1 (50.0)*	*1 (7.7)*	2 (33.3)	9 (21.4)	1 (100)	23 (23.0)	0.79
Any previous surgery/proceduresunder anaesthetic (%)	2 (28.6)	12 (27.3)	*7 (24.1)*	*0 (0)*	*5 (38.5)*	2 (33.3)	17 (40.5)	0 (0)	33 (33.0)	0.255
Previous gastrointestinal surgery/proceduresunder anaesthetic (%)	2 (28.6)	7 (15.9)	*5 (17.2)*	*0 (0)*	*2 (15.4)*	1 (16.7)	5 (11.9)	0 (0)	15 (15.0)	0.758
Previous non-gastrointestinalsurgery/procedures under anaesthetic (%)	1 (14.3)	8 (18.2)	*4 (13.8)*	*0 (0)*	*4 (30.8)*	1 (16.7)	15 (35.7)	0 (0)	25 (25.0)	0.089
**Neonatal History**										
Vaginal delivery (%)	7 (100)	35 (79.5)	*24 (82.8)*	*1 (50.0)*	*10 (76.9)*	4 (66.7)	35 (83.3)	1 (100)	82 (82.0)	0.784
Breastfed initially (%)	3 (42.9)	19 (43.2)	*15 (51.7)*	*1 (50.0)*	*3 (23.1)*	2 (33.3)	13 (31.0)	0 (0)	37 (37.0)	0.265
**Drug History**										
Previous antibiotics (%)	6 (85.7)	40 (90.9)	*27 (93.1)*	*2 (100)*	*11 (84.6)*	5 (83.3)	39 (92.9)	1 (100)	91 (91.0)	1.0
Previous steroids (%)	0 (0)	3 (6.8)	*3 (10.3)*	*0 (0)*	*0 (0)*	1 (16.7)	3 (7.1)	1 (100)	8 (8.0)	1.0
Previous acid suppression (%)	1 (14.3)	11 (25.0)	*8 (27.6)*	*0 (0)*	*3 (23.1)*	2 (33.3)	11 (26.2)	0 (0)	25 (25.0)	1.0
**Social History**										
Ethnicity white UK (%)	7 (100)	41 (93.2)	*26 (89.7)*	*2 (100)*	*13 (100)*	6 (100)	40 (95.2)	1 (100)	95 (95.0)	1.0
Smoking at home (%)	4 (57.1)	7 (15.9)	*5 (17.2)*	*0 (0)*	*2 (15.4)*	4 (66.7)	13 (31.0)	0 (0)	28 (28.0)	0.128
Pets at home (%)	7 (100)	32 (72.7)	*23 (79.3)*	*2 (100)*	*7 (53.8)*	5 (83.3)	32 (76.2)	1 (100)	77 (77.0)	1.0
Total number	7	44	*29*	*2*	*13*	6	42	1	100	

**Table 2:** BISCUIT Study Demographic and Numerical Clinical Data.

	Eosinophilic control	IBD	*Crohn’s* *disease*	*IBD-type unspecified*	*Ulcerative* *colitis*	Non-specific inflamednon-IBD	Normal colon control	Proto-IBD	All Recruits	IBD vs. Normal Colon Controlt-Test (2-tailed)
Total number	7	44	*29*	*2*	*13*	6	42	1	100	
Age (years)	10.3 (+/−3.6)	11.9 (+/−2.9)	*11.9 (+/−3.0)*	*12.0 (+/−2.0)*	*11.7 (+/−2.9)*	7.9 (+/−5.3)	10.6 (+/−3.5)	8.2	11.0 (+/−3.5)	0.067
Height Z-score	−0.67 (+/−0.89)	−0.20 (+/−1.28)	−*0.42 (+/−1.27)*	*0.26 (+/−0.11)*	*0.22 (+/−1.32)*	0.72 (+/−1.40)	0.08 (+/−1.16)	0.98	−0.02 (+/−1.23)	0.285
Weight Z-score	−0.15 (+/−0.84)	−**0.44 (+/−1.29)**	−*0.70 (+/−1.39)*	*0.16 (+/−0.27)*	*0.05 (+/−1.01)*	0.67 (+/−0.81)	**0.77 (+/−1.90)**	0.40	0.16 (+/−1.62)	**0.001**
BMI Z-Score	0.28 (+/−0.97)	−**0.53 (+/−1.39)**	−*0.74 (+/−1.46)*	*0.04 (+/−0.37)*	−*0.15 (+/−1.29)*	0.48 (+/−1.03)	**0.82 (+/−1.70)**	−0.27	0.15 (+/−1.60)	**<0.001**
Symptom duration (months)	41.9 (+/−60.6)	**9.2 (+/−12.6)**	*9.3 (+/−14.0)*	*16.0 (+/−11.3)*	*7.7 (+/−9.7)*	19.2 (+/−6.3)	**23.3 (+/−20.4)**	18	18.1 (+/−23.5)	**<0.001**
Haemoglobin (g/dl)	12.5 (+/−1.6)	**11.6 (+/−1.6)**	*11.6 (+/−1.4)*	*11.5 (+/−2.7)*	*11.8 (+/−2.1)*	11.8 (+/−1.1)	**13.4 (+/−1.5)**	12.7	12.4 (+/−1.7)	**<0.001**
White cell count (x10^9^/l)	8.4 (+/−1.0)	**9.2 (+/−3.3)**	*9.1 (+/−3.2)*	*6.1 (+/−2.2)*	*9.9 (+/−3.7)*	6.9 (+/−0.6)	**7.2 (+/−2.9)**	10.8	8.3 (+/−3.1)	**0.007**
Platelet count (x10^9^/l)	330.5 (+/−84.9)	**439.0 (+/−161.0)**	*461.9 (+/−159.0)*	*293.5 (+/−47.4)*	*407.8 (+/−167.6)*	309.3 (+/−46.5)	**300.7 (+/−69.6)**	338	370.3 (+/−138.7)	**<0.001**
C-reactive Protein (g/dl)	5.7 (+/−3.4)	**21.8 (+/−25.0)**	*26.0 (+/−28.2)*	*4.5 (+/−2.1)*	*13.6 (+/−)11.6*	4.0 (+/−3.4)	**5.8 (+/−5.7)**	6	13.3 (+/−19.3)	**<0.001**
Albumin (g/dl)	43.8 (+/−5.6)	**35.9 (+/−6.9)**	*34.8 (+/−6.8)*	*39.5 (+/−6.4)*	*38.2 (+/−7.2)*	42.0 (+/−5.5)	**44.5 (+/−2.9)**	N/A	40.1 (+/−6.9)	**<0.001**
Gestation at birth (weeks)	37.9 (+/−2.0)	39.8 (+/−1.6)	*39.9 (+/−1.5)*	*40.3 (+/−0.4)*	*39.4 (+/−1.9)*	39.6 (+/−0.9)	39.6 (+/−2.4)	40	39.6 (+/−2.0)	0.748
Birth weight (Kg)	2.84 (+/−0.37)	3.54 (+/−0.60)	*3.50 (+/−0.54)*	*3.53 (+/−0.66)*	*3.66 (+/−0.74)*	3.44 (+/−0.41)	3.30 (+/−0.59)	3.69	3.39 (+/−0.60)	0.073
Age at weaning (months)	6.1 (+/−2.4)	4.6 (+/−1.2)	*4.6 (+/−1.2)*	*6.0*	*4.3 (+/−1.3)*	5.5 (+/−0.9)	4.5 (+/−1.3)	5	4.7 (+/−1.4)	0.895
Total number	7	44	*29*	*2*	*13*	6	42	1	100	

Comparisons of clinical data are shown in **Table 1** and **Table 2**. Histological gastritis was more common in the IBD cohort than normal colon controls who underwent gastroscopy (p<0.001; **Table 1**). Conversely, histological identification of *H. pylori* was higher in the normal colon controls and absent in the IBD cohort (p = 0.035; **Table 1**).

**Figure 2:**
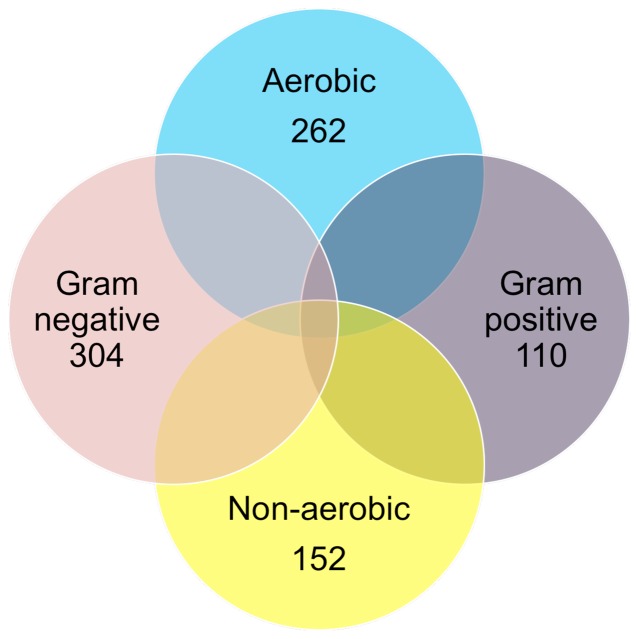
Basic phenotypic assessment of 414 bacterial isolates obtained from the paediatric colonic mucosa. 129 were both Gram-negative and non-aerobic, of which 114 were formally identified by sequencing.

Of 555 attempted bacterial subcultures, 494 demonstrated some growth within 7 days, with 414 yielding sufficient growth to allow Gram-staining and aerobic subculture challenge to be completed (**Figure 2**). 129 bacterial isolates met the requirements for further identification (Gram-negative but failed aerobic subculture). Of these, 114 yielded sufficient growth for DNA based identification. 112 were confirmed as Gram-negative microaerophilic bacteria after formal sequence identification. The formal identities of these 112, including 73 distinct patient isolates, are presented in **Table 3**. The identities of the remaining two isolates matched two separate Gram-positive species (*Bifidobacterium longum* and *Enterococcus faecalis*) hence they were removed.

**Table 3:** Bacterial Isolates Identified based on 16S rDNA sequencing.

Isolate	Number of SubculturesObtained (May include duplicates from same patientderived fromdifferent growth media)	DistinctPatientIsolates	Source	16SSequenceLength	PercentageSimilarityon BLAST
*Alistipes finegoldii*	1	1	IBD-type unspecified	515 bp	100%
*Bacteroides caccae*	3	2	Non-specific inflamednon-IBD (1), IBD-typeunspecified (1)	513–795 bp	99%
*Bacteroides dorei*	1	1	Normal colon control	684 bp	100%
*Bacteroides fragilis*	1	1	Crohn’s disease	526 bp	99%
*Bacteroides nordii*	1	1	Normal colon control	566 bp	99%
*Bacteroides ovatus*	2	2	Normal colon control (2)	433 bp	99%
*Bacteroides salyersiae*	2	1	IBD-type unspecified (2)	697 bp, 823 bp	99%
*Bacteroides thetaiotaomicron*	1	1	Crohn’s disease	415 bp	100%
*Bacteroides uniformis*	1	1	Normal colon control	503 bp	99%
*Butyricimonas virosa*	2	2	Normal colon control (1), IBD-type unspecified (1)	569 bp, 676 bp	98–99%
*Campylobacter concisus*	6	3	Crohn’s disease (2), Ulcerative colitis (1)	1357–1423 bp	99–100%
*Campylobacter curvus*	1	1	Normal colon control	1537 bp	99%
*Campylobacter lari*	1	1	Normal colon control	647 bp	100%
*Campylobacter rectus*	1	1	Normal colon control	401 bp	100%
*Campylobacter showae*	6	3	Normal colon control (2), Crohn’s disease (1)	1325–1422 bp	99%
*Eikenella corrodens*	2	1	Crohn’s disease	776–802 bp	99–100%
*Haemophilus parainfluenzae*	8	7	Crohn’s disease (3), Ulcerative colitis (2),Normal colon control (2)	455–807 bp	99–100%
*Odoribacter splanchnicus*	1	1	Eosinophilic control	819 bp	99%
*Parabacteroides distasonis*	7	7	Normal colon control (6),Ulcerative colitis (1)	412–786 bp	99%
*Sutterella wadsworthensis*	61	32	Normal colon control (11), Crohn’sdisease (8), Ulcerative colitis (6),Eosinophilic control (3),Non-specific inflamed non-IBD (2),IBD-type unspecified (1), Proto-IBD (1)	411–1423 bp	97–100%
*Terrahaemophilus aromaticivorans*	3	3	Crohn’s disease (1), Ulcerativecolitis (1), Normal colon control (1)	554 672 bp	99%
**Total**	112	73			

PCR prevalence data and *Helicobacter pylori* serology results for each of the phenotypic categories are shown in **Table 4** and for each of the subjects in **Table S2**. There was no significant difference in PCR prevalence for *Helicobacter, Campylobacter* or *Sutterella wadsworthensis* between the IBD cohort and normal colon controls (Table 4). No *H. pylori* seropositive subject was positive for *Helicobacter* PCR and *vice-versa*. **Table 5** documents *Campylobacter* PCR sequencing data to species-level stratified by clinical phenotype. It is apparent from the data that individuals can harbour multiple distinct species. Of the 72 positive subjects, 44 had a single *Campylobacter* identified with two species being identified in 17 and three species in the remaining 11 (**Table 6**). *Campylobacter curvus*, *Campylobacter gracilis* and *Campylobacter ureolyticus* were never identified in isolation. The *Helicobacter* sequencing data was far less complex with only 12 subjects yielding positive PCR product. Of these, 5 were from normal colon controls, 5 from IBD (4 CD, 1 UC) and 2 were from eosinophilic controls. After sequencing the PCR product, 8 of the 12 (4 normal colon controls, 2 IBD- both CD, 2 eosinophilic controls) were identified as *Wolinella succinogenes*, another Epsilonproteobacteria and member of the Helicobacteraceae. The four remaining *Helicobacter* positive results were not identifiable by direct sequencing and underwent cloning and sequencing analysis. This revealed the presence of both *W. succinogenes* and *Helicobacter brantae* (from a CD patient) and confirmed the presence of *Helicobacter hepaticus* from a second CD patient. The two remaining patient samples remained unidentifiable despite repeated cloning attempts.

**Table 4:** PCR Prevalence and *Helicobacter pylori* Serology Data from BISCUIT Study.

	*Helicobacter pylori* Serology Positive	*Helicobacter* Genus PCR Positive	*Campylobacter* Genus PCR Positive	*Sutterella wadsworthensis* PCR Positive	Total Subjects
**IBD**	**1 (2.3%)**	**5 (11.4%)**	**33 (75.0%)**	**36 (81.8%)**	**44**
(Crohn’s disease)	0 (0%)	4 (13.8%)	22 (75.9%)	23 (79.3%)	29
(Ulcerative colitis)	1 (7.7%)	1 (7.7%)	9 (69.2%)	11 (84.6%)	13
(IBD-type unspecified)	0 (0%)	0 (0%)	2 (100%)	2 (100%)	2
**Normal colon control**	**6 (14.3%)**	**5 (11.9%)**	**32 (76.2%)**	**30 (71.4%)**	**42**
Eosinophilic control	1 (14.3%)	2 (28.6%)	4 (57.1%)	7 (100%)	7
Non-specific inflamed non-IBD	0 (0%)	0 (0%)	3 (50.0%)	5 (83.3%)	6
Proto-IBD	0 (0%)	0 (0%)	0 (0%)	1 (100%)	1
**Total**	**8 (8.0%)**	**12 (12.0%)**	**72 (72.0%)**	**79 (79.0%)**	**100**
IBD vs. Normal colon control by Fisher’sexact test (2-sided, n = 86)	p = 0.055	p = 1.00	p = 1.00	p = 0.312	

**Table 5:** *Campylobacter* Results Obtained Through Sequence Analysis.

	*Campylobacter Genus PCR Positive*	*Campylobacter concisus*	*Campylobacter curvus*	*Campylobacter gracilis*	*Campylobacter hominis*	*Campylobacter lari*	*Campylobacter rectus*	*Campylobacter showae*	*Campylobacter ureolyticus*	TotalSubjects
**IBD**	**33 (75.0%)**	17 (38.6%)	2 (4.5%)	1 (2.3%)	15 (34.1%)	1 (2.3%)	1 (2.3%)	14 (31.8%)	0 (0%)	**44**
(Crohn’s disease)	22 (75.9%)	13 (44.8%)	2 (6.9%)	1 (3.4%)	9 (31.0%)	1 (3.4%)	0 (0%)	9 (31.0%)	0 (0%)	29
(Ulcerative colitis)	9 (69.2%)	4 (30.8%)	0 (0%)	0 (0%)	5 (38.5%)	0 (0%)	0 (0%)	5 (38.5%)	0 (0%)	13
(IBD-type unspecified)	2 (100%)	0 (0%)	0 (0%)	0 (0%)	1 (50.0%)	0 (0%)	1 (50.0%)	0 (0%)	0 (0%)	2
**Normal colon control**	**32 (76.2%)**	16 (38.1%)	3 (7.1%)	2 (4.8%)	14 (33.3%)	0 (0%)	4 (9.5%)	9 (21.4%)	2 (4.8%)	**42**
Eosinophilic control	4 (57.1%)	2 (28.6%)	0 (0%)	0 (0%)	1 (14.3%)	0 (0%)	1 (14.3%)	3 (42.9%)	0 (0%)	7
Non-specific inflamed non-IBD	3 (50.0%)	2 (33.3%)	0 (0%)	0 (0%)	1 (16.7%)	0 (0%)	0 (0%)	0 (0%)	0 (0%)	6
Proto-IBD	0 (0%)	0 (0%)	0 (0%)	0 (0%)	0 (0%)	0 (0%)	0 (0%)	0 (0%)	0 (0%)0	1
**Total**	**72 (72.0%)**	37 (37.0%)	5 (5.0%)	3 (3.0%)	31 (31.0%)	1 (1.0%)	6 (6.0%)	26 (26.0%)	2 (2.0%)	**100**

**Table 6:** Campylobacter Sequencing Results at Species-Level by Number of Species per Subject.

Single Species	Number of Subjects	Source
*Campylobacter concisus*	18	Normal colon control (6), Crohn’s disease (8), Ulcerative colitis (2), Non-specific inflamed non-IBD (2)
*Campylobacter hominis*	10	Normal colon control (5), Crohn’s disease (1), Ulcerative colitis (2), Non-specific inflamed non-IBD (1), IBD-type unspecified (1)
*Campylobacter lari*	1	Crohn’s disease (1)
*Campylobacter rectus*	5	Normal colon control (3), Eosinophilic control (1), IBD-type unspecified (1)
*Campylobacter showae*	10	Normal colon control (5), Crohn’s disease (2), Ulcerative colitis (2), Eosinophilic control (1)
Total	44	
Two Species	Number of Subjects	Source
*Campylobacter concisus*+*Campylobacter curvus*	2	Normal colon control (1), Crohn’s disease (1)
*Campylobacter concisus*+*Campylobacter hominis*	5	Normal colon control (4), Crohn’s disease (1)
*Campylobacter concisus*+*Campylobacter showae*	2	Normal colon control (1), Eosinophilic control (1)
*Campylobacter gracilis*+*Campylobacter hominis*	1	Crohn’s disease (1)
*Campylobacter gracilis*+*Campylobacter showae*	1	Normal colon control (1)
*Campylobacter hominis*+*Campylobacter showae*	5	Crohn’s disease (4), Ulcerative colitis (1)
*Campylobacter hominis*+*Campylobacter ureolyticus*	1	Normal colon control (1)
Total	17	
Three Species	Number of Subjects	Source
*Campylobacter concisus*+*Campylobacter curvus*+*Campylobacter showae*	2	Normal colon control (1), Crohn’s disease (1)
*Campylobacter concisus*+*Campylobacter curvus*+*Campylobacter hominis*	1	Normal colon control (1)
*Campylobacter concisus*+*Campylobacter gracilis*+*Campylobacter hominis*	1	Normal colon control (1)
*Campylobacter concisus*+*Campylobacter hominis*+*Campylobacter rectus*	1	Normal colon control (1)
*Campylobacter concisus*+*Campylobacter hominis*+*Campylobacter showae*	5	Crohn’s disease (2), Ulcerative colitis (2), Eosinophilic control (1)
*Campylobacter hominis*+*Campylobacter showae*+*Campylobacter ureolyticus*	1	Normal colon control (1)
Total	11	

## Discussion

This study comprehensively describes the microaerophilic microbiota of the paediatric colon with specific reference to untreated, new-onset paediatric IBD and also those with a normal colon. Our main findings are of a high molecular prevalence and culture recovery rate of unusual *Campylobacter* species and *S. wadsworthensis* and of a low molecular prevalence of Helicobacteraceae. There was no difference in the prevalence of microaerophilic species between IBD patients and controls. We acknowledge that the microaerophilic microbiota comprises a relatively small proportion of the bacterial community present within the colon, with the majority of species being obligate anaerobes. Evidence supporting a role for these microaerophilic species in IBD cannot however be ignored and they are therefore worthy of targeted study.

The possibility that *Helicobacter* species may be involved in IBD pathogenesis is an intriguing one that has been the subject of many studies and much debate [17]–[26]. The earliest observation that *H. pylori* seropositivity is negatively associated with IBD [39] was not directly replicated in this study, however in our recruits undergoing concurrent upper gastrointestinal endoscopy, microscopic evidence of *H. pylori* was entirely absent from the IBD cohort and significantly higher in normal colon controls. *C. concisus* is an organism that has generated significant interest following culture recovery from the colon of children with CD, with subsequent work describing the adherent, invasive and pro-inflammatory capabilities of the organism [27], [29]. Other authors have suggested that the organism may be increased in IBD against controls [28], [30], [31], yet our data contradicts this finding by demonstrating a comparable prevalence between the two groupings. Our data are the first to specifically address these organisms at the onset of IBD. The low prevalence of Helicobacteraceae and equivalent prevalence of *C. concisus* at the onset of IBD which we have shown makes it unlikely that these organisms have a role in disease pathogenesis in children; nevertheless their identification in the colon of subjects with established disease in other studies suggests that roles within disease chronicity may still be possible.

We have described a surprisingly diverse and prevalent colonisation of the paediatric colon with unusual *Campylobacter* species, including the possibility of up to three distinct species co-existing in close proximity in the same individual. The importance of these *Campylobacter* in paediatric health and disease warrants further consideration, particularly given the unquestionable pathogenicity of *Campylobacter jejuni* and *Campylobacter coli*, the two most commonly identified representatives of the genus in paediatric faecal samples [40]. Our data suggest a more diverse and prevalent colonic colonisation with *Campylobacter* species than previously reported. This finding may be a direct reflection of the sampling bias introduced by studying faeces alone which is known to represent a distinct ecosystem [41]. We have shown that unusual *Campylobacter* species can be identified in the colon of 7/10 children and cultured successfully from 8/100. Additional studies are required to increase the culture yield for these organisms and to characterise individual species and further outline their role in health and disease.

*Sutterella wadsworthensis* is an organism that has rarely been discussed in the literature, having first been described as a potential gastrointestinal pathogen in 1996 [42]. We recently examined the molecular prevalence of this organism in an adult study including those with UC and those with a normal colon and found a similar and high prevalence in both groups [37]. Phenotypic and genotypic comparison of isolates suggested no difference between the two clinical groups. We suggested therefore that *S. wadsworthensis* is likely a common intestinal commensal. A recent paper on 32 children has however linked *S. wadsworthensis* to autism [43], generating considerable discussion in the process [44], [45]. We again find within the paediatric population, that this organism is commonly identified and easily recovered from biopsies by culture. Given the high prevalence (79% overall) of this organism in our whole cohort, we consider it unlikely that it is specific to the autistic intestine as has been suggested. This of course does not exclude differential immunological reaction to the organism within autistic children, which requires further exploration.

The limitations of this study cohort have been discussed previously [32] and will be repeated here briefly. All subjects received stimulant bowel preparation before colonoscopy. Although this may have altered the bacteria within the colonic mucosa, the treatment was given to all and would likely act equivalently between groups. A study where children undergo colonoscopy under general anaesthetic without bowel preparation would be unethical. The controls in this study were all children undergoing colonoscopy for gastrointestinal symptoms. There were therefore no strictly “healthy” controls. We have tried to address this by describing our subcategorisation of recruits in detail and selecting only those with a macroscopically and microscopically normal colon as our main control group.

This study rejects a role for the microaerophilic bacteria *Helicobacter*, *Campylobacter* and *S. wadsworthensis* at the initiation of paediatric IBD, however hypothesis-free analysis of a subgroup of the same study using pyrosequencing has shown that differences in the IBD microbiota are apparent at the onset of disease. Of particular interest to the culture results from this study, we have shown that *Parabacteroides* appear to be significantly reduced in UC against normal colon controls [32]. This appears to be reflected further in our culture recovery rate of *Parabacteroides distasonis* reported here (6 isolates derived from normal colon controls against a single UC isolate). This observation would fit with the discoveries that *P. distasonis* antigens can attenuate murine colitis and are specifically recognised by colonic T regulatory cells [46], [47]. *P. distasonis* might be suitable for consideration as a probiotic bacterium for topical colonic treatment in UC.

### Conclusion

This study has provided novel data describing a hitherto unrecognised high prevalence and diversity of unusual *Campylobacter* species and a high prevalence of *S. wadsworthensis* in the paediatric colon. We have also shown a low prevalence of organisms within the Helicobacteraceae. Although we have not demonstrated any organisms of likely significance to IBD pathogenesis, we have explored two likely candidate genera specifically at the onset of disease and demonstrated that their involvement in disease initiation is unlikely. Our data on *S. wadsworthensis* refutes the suggestion that this organism is specific to the paediatric autistic intestine, and alongside our previous work, suggests this organism is a common intestinal commensal. Our isolates of *P. distasonis* from the normal paediatric colon might be suitable for consideration as probiotics. We have shown that a targeted culture and molecular microbiology study in the paediatric population can demonstrate surprising results and offer a high yield for the enumeration of unusual and rarely described organisms.

## Supporting Information

Table S1 PCR Primers Used in This Study.
(DOCX)

Table S2 BISCUIT Patient Cohort PCR and *H. pylori* Serology Results for Individual Recruits with Phenotype.
(DOCX)
