# Supplementary material for: The Microaerophilic Microbiota of De-Novo Paediatric Inflammatory Bowel Disease: The BISCUIT Study
Source: PLoS One. 2013 Mar 12;8(3):e58825. doi: 10.1371/journal.pone.0058825 (PMC3595230; doi:10.1371/journal.pone.0058825)
Supplement: Table S1 — PCR Primers Used in This Study. (DOCX) [file pone.0058825.s001.docx]

**Table S1:** PCR Primers Used in This Study

| Primer Set | Sequence | Reference |
| --- | --- | --- |
| Universal Bacterial  27F/1492R | 27F:5’- AGA GTT TGA TCM TGG CTC AG – 3’ | ^38^ |
|  | 1492R: 5’ - ACG GCT ACC TTG TTA CGA CTT – 3’ |  |
| *Helicobacter* genus  C05/C97 followed by C98/1067R | C05: 5’ - ACT TCA CCC CAG TCG CTG – 3’ | ^25,48,49^ |
|  | C97: 5’ - GCT ATG ACG GGT ATC C – 3’ |  |
|  | C98: 5’ - TGG TGT AGG GGT AAA ATC C – 3’ |  |
|  | 1067R: 5’ - Gcc gtg cag cac ctg ttt tca – 3’ |  |
| *Campylobacter* genus  C412F/C1228R | C412F: 5’ - GGA TGA CAC TTT TCG GAG C – 3’ | ^30,50^ |
|  | C1228R 5’ - CAT GTA GCA CGT GTG TC – 3’ |  |
| *Sutterella wadsworthensis*  SWF/SWR | SWF: 5’- GAC GAA AAG GGA TGC GAT AA – 3’ | ^37^ |
|  | SWR: 5’- CTG GCA TGT CAA GGC TAG GT- 3’ |  |
