# Supplementary material for: The Microaerophilic Microbiota of De-Novo Paediatric Inflammatory Bowel Disease: The BISCUIT Study
Source: PLoS One. 2013 Mar 12;8(3):e58825. doi: 10.1371/journal.pone.0058825 (PMC3595230; doi:10.1371/journal.pone.0058825)
Supplement: Table S2 — BISCUIT Patient Cohort PCR and H. pylori Serology Results for Individual Recruits with Phenotype. (DOCX) [file pone.0058825.s002.docx]

**Table S2:** BISCUIT Patient Cohort PCR and *H. pylori* Serology Results for Individual Recruits with Phenotype

| Diagnostic Category | Biopsy Site | Sex | Paris Age | Montreal | Paris | Height Z-score | Weight Z-score | BMI  Z-score | *H. pylori* Serology | *Helicobacter* Genus PCR | *Campylobacter* Genus PCR | *S. wadsworthensis* PCR |
| --- | --- | --- | --- | --- | --- | --- | --- | --- | --- | --- | --- | --- |
| Normal colon control | Rectum | Male | 0-10 years |  |  | -0.4 | -0.7 | -0.7 | Negative | Positive | Positive | Positive |
| Normal colon control | Rectum | Male | 10-17 years |  |  | -1.4 | -1.3 | -0.7 | Positive | Negative | Positive | Positive |
| Normal colon control | Caecum | Male | 0-10 years |  |  | 0.9 | 1.8 | 2.0 | Negative | Negative | Positive | Positive |
| Normal colon control | Rectum | Male | 10-17 years |  |  | -0.5 | 1.6 | 2.4 | Negative | Negative | Positive | Positive |
| Normal colon control | Rectum | Male | 0-10 years |  |  | -4.0 | 10.6 | 8.9 | Negative | Negative | Positive | Positive |
| Normal colon control | Rectum | Male | 10-17 years |  |  | 0.7 | 0.3 | -0.2 | Negative | Negative | Positive | Negative |
| Normal colon control | Rectum | Male | 0-10 years |  |  | 0.3 | 0.9 | 1.1 | Negative | Negative | Positive | Positive |
| Normal colon control | Rectum | Male | 10-17 years |  |  | 0.9 | 1.6 | 1.7 | Negative | Positive | Positive | Positive |
| Normal colon control | Sigmoid | Male | 0-10 years |  |  | 1.0 | 1.5 | 1.4 | Negative | Positive | Positive | Positive |
| Normal colon control | Rectum | Male | 10-17 years |  |  | 0.1 | -0.1 | -0.1 | Negative | Positive | Positive | Positive |
| Normal colon control | Caecum | Male | 0-10 years |  |  | 2.2 | 2.2 | 1.8 | Negative | Positive | Positive | Positive |
| Normal colon control | Sigmoid | Female | 10-17 years |  |  | -0.1 | 0.9 | 1.2 | Negative | Negative | Positive | Positive |
| Normal colon control | Sigmoid | Male | 0-10 years |  |  | -0.3 | -0.3 | -0.1 | Negative | Negative | Negative | Positive |
| Normal colon control | Sigmoid | Male | 10-17 years |  |  | 1.8 | 2.3 | 2.2 | Negative | Negative | Positive | Positive |
| Normal colon control | Rectum | Male | 0-10 years |  |  | -1.0 | 0.7 | 1.7 | Positive | Negative | Negative | Positive |
| Normal colon control | Sigmoid | Male | 10-17 years |  |  | 0.9 | -0.2 | -1.2 | Negative | Negative | Positive | Positive |
| Normal colon control | Sigmoid | Female | 0-10 years |  |  | -0.1 | 0.1 | 0.2 | Negative | Negative | Negative | Positive |
| Normal colon control | Sigmoid | Female | 10-17 years |  |  | 0.1 | 0.8 | 0.9 | Negative | Negative | Negative | Positive |
| Normal colon control | Sigmoid | Male | 10-17 years |  |  | 0.0 | 0.3 | 0.4 | Negative | Negative | Positive | Positive |
| Normal colon control | Sigmoid | Male | 10-17 years |  |  | 0.8 | -0.2 | -1.0 | Positive | Negative | Positive | Positive |
| Normal colon control | Rectum | Male | 10-17 years |  |  | 0.7 | 1.9 | 2.1 | Positive | Negative | Negative | Positive |
| Normal colon control | Rectum | Male | 10-17 years |  |  | 1.1 | 0.2 | -0.6 | Negative | Negative | Negative | Positive |
| Normal colon control | Sigmoid | Male | 10-17 years |  |  | -0.7 | -0.3 | 0.0 | Positive | Negative | Positive | Negative |
| Normal colon control | Caecum | Male | 0-10 years |  |  | 0.0 | 0.2 | 0.2 | Negative | Negative | Negative | Negative |
| Normal colon control | Caecum | Female | 10-17 years |  |  | 0.1 | -0.5 | -0.7 | Positive | Negative | Positive | Positive |
| Normal colon control | Sigmoid | Male | 10-17 years |  |  | 1.3 | 2.2 | 2.0 | Negative | Negative | Positive | Negative |
| Normal colon control | Sigmoid | Male | 0-10 years |  |  | -0.6 | -0.9 | -0.9 | Negative | Negative | Negative | Positive |
| Normal colon control | Sigmoid | Female | 10-17 years |  |  | 2.1 | 1.7 | 1.0 | Negative | Negative | Positive | Negative |
| Normal colon control | Sigmoid | Male | 0-10 years |  |  | 0.5 | 2.2 | 2.6 | Negative | Negative | Positive | Positive |
| Normal colon control | Sigmoid | Male | 0-10 years |  |  | -0.3 | 0.5 | 1.0 | Negative | Negative | Positive | Negative |
| Normal colon control | Sigmoid | Female | 0-10 years |  |  | -1.8 | -0.8 | 0.7 | Negative | Negative | Positive | Negative |
| Normal colon control | Sigmoid | Male | 10-17 years |  |  | 0.5 | 0.8 | 0.8 | Negative | Negative | Positive | Negative |
| Normal colon control | Sigmoid | Male | 10-17 years |  |  | 0.4 | 1.2 | 1.3 | Negative | Negative | Positive | Positive |
| Normal colon control | Sigmoid | Male | 0-10 years |  |  | 0.9 | 0.9 | 0.5 | Negative | Negative | Positive | Positive |
| Normal colon control | Sigmoid | Female | 0-10 years |  |  | 0.3 | 1.2 | 1.4 | Negative | Negative | Negative | Positive |
| Normal colon control | Sigmoid | Male | 10-17 years |  |  | -1.5 | -0.3 | 0.9 | Negative | Negative | Positive | Negative |
| Normal colon control | Sigmoid | Female | 10-17 years |  |  | -0.9 | -0.6 | -0.3 | Negative | Negative | Negative | Positive |
| Normal colon control | Sigmoid | Male | 10-17 years |  |  | 0.0 | 1.1 | 1.5 | Negative | Negative | Positive | Negative |
| Normal colon control | Sigmoid | Female | 0-10 years |  |  | 1.2 | 0.6 | -0.1 | Negative | Negative | Positive | Positive |
| Normal colon control | Sigmoid | Male | 10-17 years |  |  | -0.4 | -0.3 | 0.0 | Negative | Negative | Positive | Negative |
| Normal colon control | Sigmoid | Male | 10-17 years |  |  | 0.5 | 1.2 | 1.3 | Negative | Negative | Positive | Negative |
| Normal colon control | Sigmoid | Male | 10-17 years |  |  | -2.0 | -2.8 | -2.2 | Negative | Negative | Positive | Positive |
| Eosinophilic control | Rectum | Female | 10-17 years |  |  | 0.0 | 1.0 | 1.3 | Negative | Positive | Negative | Positive |
| Eosinophilic control | Rectum | Female | 10-17 years |  |  | -0.9 | -0.7 | -0.3 | Negative | Positive | Positive | Positive |
| Eosinophilic control | Rectum | Male | 10-17 years |  |  | 0.3 | -0.1 | -0.4 | Negative | Negative | Positive | Positive |
| Eosinophilic control | Caecum | Male | 10-17 years |  |  | -0.7 | 1.0 | 1.7 | Negative | Negative | Positive | Positive |
| Eosinophilic control | Sigmoid | Female | 0-10 years |  |  | -1.4 | -1.1 | -0.3 | Negative | Negative | Negative | Positive |
| Eosinophilic control | Caecum | Male | 0-10 years |  |  | -2.1 | -0.8 | 0.7 | Positive | Negative | Positive | Positive |
| Eosinophilic control | Sigmoid | Female | 0-10 years |  |  | 0.1 | -0.5 | -0.8 | Negative | Negative | Negative | Positive |
| Non-specific inflamed non-IBD | Rectum | Female | 10-17 years |  |  | 1.1 | -0.1 | -0.7 | Negative | Negative | Negative | Positive |
| Non-specific inflamed non-IBD | Sigmoid | Male | 0-10 years |  |  | -1.4 | 0.2 | 1.5 | Negative | Negative | Positive | Positive |
| Non-specific inflamed non-IBD | Rectum | Female | 10-17 years |  |  | 2.0 | 1.2 | 0.3 | Negative | Negative | Negative | Positive |
| Non-specific inflamed non-IBD | Sigmoid | Female | 0-10 years |  |  | 2.4 | 1.7 | 0.6 | Negative | Negative | Positive | Positive |
| Non-specific inflamed non-IBD | Sigmoid | Female | 0-10 years |  |  | 0.4 | -0.2 | -0.6 | Negative | Negative | Negative | Negative |
| Non-specific inflamed non-IBD | Sigmoid | Male | 0-10 years |  |  | -0.1 | 1.3 | 1.8 | Negative | Negative | Positive | Positive |
| Proto-IBD | Descending | Male | 0-10 years |  |  | 1.0 | 0.4 | -0.3 | Negative | Negative | Negative | Positive |
| Crohn's disease | Rectum | Male | 10-17 years | L3+L4, B1 | L3+L4a, B1 | 1.8 | 1.0 | 0.1 | Negative | Positive | Negative | Positive |
| Crohn's disease | Transverse | Female | 10-17 years | L3+L4, B1 | L3+L4a, B1 | 0.4 | 0.1 | -0.2 | Negative | Positive | Positive | Positive |
| Crohn's disease | Descending | Female | 10-17 years | L3+L4, B2 | L3+L4a, B2 | -1.1 | -1.4 | -1.3 | Negative | Negative | Positive | Positive |
| Crohn's disease | Caecum | Male | 10-17 years | L1, B2 | L1, B2 | -0.3 | -0.9 | -1.2 | Negative | Negative | Positive | Positive |
| Crohn's disease | Rectum | Female | 10-17 years | L2, B1 | L2, B1 | 1.9 | 1.6 | 1.2 | Negative | Negative | Positive | Negative |
| Crohn's disease | Caecum | Male | 10-17 years | L3, B2 | L3, B2 | -0.1 | 0.1 | 0.3 | Negative | Negative | Negative | Positive |
| Crohn's disease | Rectum | Female | 10-17 years | L3, B2 | L3, B2 | 0.4 | -1.0 | -1.7 | Negative | Positive | Positive | Positive |
| Crohn's disease | Rectum | Male | 10-17 years | L3+L4, B1 | L3+L4a, B1 | -0.7 | -2.5 | -3.7 | Negative | Positive | Positive | Positive |
| Crohn's disease | Sigmoid | Male | 10-17 years | L3+L4, B1 | L3+L4a, B1 | -2.5 | -2.2 | -0.9 | Negative | Negative | Positive | Positive |
| Crohn's disease | Sigmoid | Male | 10-17 years | L2+L4, B1 | L2+L4a, B1 | 0.8 | 2.6 | 2.8 | Negative | Negative | Negative | Positive |
| Crohn's disease | Sigmoid | Female | 0-10 years | L3, B1 | L3, B1 | -1.8 | -1.1 | 0.0 | Negative | Negative | Positive | Positive |
| Crohn's disease | Sigmoid | Male | 10-17 years | L2, B1 | L2, B1 | 1.6 | 1.2 | 0.7 | Negative | Negative | Positive | Positive |
| Crohn's disease | Caecum | Female | 0-10 years | L3, B1 | L3, B1 | -1.5 | -0.9 | -0.1 | Negative | Negative | Positive | Positive |
| Crohn's disease | Sigmoid | Female | 10-17 years | L3, B1 | L3, B1 | -1.6 | -1.8 | -1.5 | Negative | Negative | Positive | Positive |
| Crohn's disease | Descending | Male | 0-10 years | L3+L4, B1 | L3+L4a, B1 | -1.2 | -0.8 | -0.1 | Negative | Negative | Negative | Positive |
| Crohn's disease | Caecum | Male | 10-17 years | L3, B1 | L3, B1 | -2.2 | -2.0 | -1.1 | Negative | Negative | Negative | Positive |
| Crohn's disease | Sigmoid | Male | 0-10 years | L0, B3p | L0, B3p | -0.6 | 0.0 | 0.5 | Negative | Negative | Positive | Positive |
| Crohn's disease | Sigmoid | Male | 10-17 years | L3+L4, B1p | L3+L4a/b, B1p | -1.3 | -1.6 | -1.1 | Negative | Negative | Positive | Negative |
| Crohn's disease | Sigmoid | Male | 10-17 years | L3+L4, B1 | L3+L4a, B1 | 0.8 | -0.6 | -1.4 | Negative | Negative | Positive | Positive |
| Crohn's disease | Sigmoid | Male | 10-17 years | L3+L4, B1 | L3+L4a/b, B1 | -2.2 | -2.0 | -1.0 | Negative | Negative | Positive | Positive |
| Crohn's disease | Sigmoid | Male | 10-17 years | L2, B1 | L2, B1 | 0.2 | -1.2 | -2.3 | Negative | Negative | Positive | Positive |
| Crohn's disease | Sigmoid | Female | 0-10 years | L2, B1 | L2, B1 | 1.8 | 1.9 | 1.1 | Negative | Negative | Positive | Negative |
| Crohn's disease | Sigmoid | Male | 10-17 years | L2, B1 | L2, B1 | 0.1 | -0.1 | -0.3 | Negative | Negative | Negative | Negative |
| Crohn's disease | Descending | Female | 10-17 years | L3+L4, B1 | L3+L4a, B1 | 0.0 | -1.7 | -3.1 | Negative | Negative | Negative | Positive |
| Crohn's disease | Rectum | Male | 10-17 years | L3, B2 | L3, B2 | -0.9 | -0.1 | 0.4 | Negative | Negative | Positive | Positive |
| Crohn's disease | Sigmoid | Male | 10-17 years | L2, B1 | L2, B1 | -1.3 | -2.0 | -1.9 | Negative | Negative | Positive | Positive |
| Crohn's disease | Sigmoid | Male | 10-17 years | L3, B1 | L3, B1 | -0.6 | -0.8 | -0.7 | Negative | Negative | Positive | Negative |
| Crohn's disease | Transverse | Male | 10-17 years | L3, B1 | L3, B1 | -2.0 | -3.4 | -4.0 | Negative | Negative | Positive | Positive |
| Crohn's disease | Sigmoid | Male | 10-17 years | L2+L4, B1 | L2+L4a, B1 | -0.2 | -0.6 | -0.7 | Negative | Negative | Positive | Negative |
| IBD-type unspecified | Sigmoid | Male | 10-17 years | E3 | E3 | 0.2 | 0.0 | -0.2 | Negative | Negative | Positive | Positive |
| IBD-type unspecified | Descending | Female | 10-17 years | E2 | E2 | 0.3 | 0.4 | 0.3 | Negative | Negative | Positive | Positive |
| Ulcerative colitis | Rectum | Male | 0-10 years | E3 | E3 | 1.4 | 1.3 | 1.0 | Negative | Positive | Negative | Positive |
| Ulcerative colitis | Rectum | Male | 10-17 years | E3 | E4 | -1.1 | -1.6 | -1.6 | Negative | Negative | Positive | Positive |
| Ulcerative colitis | Rectum | Male | 10-17 years | E3 | E4 | 0.1 | -0.8 | -1.5 | Negative | Negative | Positive | Positive |
| Ulcerative colitis | Sigmoid | Male | 10-17 years | E2 | E2 | 1.0 | 1.8 | 1.7 | Negative | Negative | Positive | Positive |
| Ulcerative colitis | Sigmoid | Male | 10-17 years | E3 | E4 | 2.8 | 1.1 | -0.6 | Negative | Negative | Positive | Positive |
| Ulcerative colitis | Sigmoid | Female | 0-10 years | E2 | E2 | -1.7 | -0.1 | 1.0 | Negative | Negative | Positive | Positive |
| Ulcerative colitis | Sigmoid | Male | 10-17 years | E2 | E2 | -1.2 | 0.1 | 1.0 | Negative | Negative | Positive | Positive |
| Ulcerative colitis | Sigmoid | Male | 10-17 years | E3 | E4 | 0.5 | 0.5 | 0.3 | Positive | Negative | Positive | Positive |
| Ulcerative colitis | Sigmoid | Female | 0-10 years | E3 | E3 | 0.2 | 0.6 | 0.7 | Negative | Negative | Positive | Negative |
| Ulcerative colitis | Sigmoid | Male | 0-10 years | E3 | E4 | -1.4 | -0.4 | 0.5 | Negative | Negative | Negative | Positive |
| Ulcerative colitis | Sigmoid | Female | 10-17 years | E3 | E4 | 0.7 | -0.8 | -1.7 | Negative | Negative | Positive | Positive |
| Ulcerative colitis | Sigmoid | Female | 10-17 years | E2 | E2 | 1.6 | 0.2 | -0.8 | Negative | Negative | Negative | Negative |
| Ulcerative colitis | Sigmoid | Male | 10-17 years | E3 | E4 | -0.1 | -1.3 | -2.2 | Negative | Negative | Negative | Positive |
